# Subpopulations of *Staphylococcus aureus* Clonal Complex 121 Are Associated with Distinct Clinical Entities

**DOI:** 10.1371/journal.pone.0058155

**Published:** 2013-03-07

**Authors:** Kevin Kurt, Jean-Philippe Rasigade, Frederic Laurent, Richard V. Goering, Helena Žemličková, Ivana Machova, Marc J. Struelens, Andreas E. Zautner, Silva Holtfreter, Barbara Bröker, Stephen Ritchie, Sin Reaksmey, Direk Limmathurotsakul, Sharon J. Peacock, Christiane Cuny, Franziska Layer, Wolfgang Witte, Ulrich Nübel

**Affiliations:** 1 Robert Koch Institute, Wernigerode, Germany; 2 National Reference Center for Staphylococci, Faculty of Medicine, University of Lyon, Lyon, France; 3 Creighton University School of Medicine, Omaha, Nebraska, United States of America; 4 National Institute of Public Health, Prague, Czech Republic; 5 European Centre for Disease Prevention and Control, Stockholm, Sweden; 6 Institut für Medizinische Mikrobiologie, Universitätsmedizin Göttingen, Göttingen, Germany; 7 Institute of Immunology and Transfusion Medicine, Ernst Moritz Arndt University, Greifswald, Germany; 8 Department of Molecular Medicine and Pathology, University of Auckland, Auckland, New Zealand; 9 Angkor Hospital for Children, Siem Reap, Cambodia; 10 Faculty of Tropical Medicine, Mahidol University, Bangkok, Thailand; 11 Department of Medicine, University of Cambridge, Cambridge, United Kingdom; University of Liverpool, United Kingdom

## Abstract

We investigated the population structure of *Staphylococcus aureus* clonal complex CC121 by mutation discovery at 115 genetic housekeeping loci from each of 154 isolates, sampled on five continents between 1953 and 2009. In addition, we pyro-sequenced the genomes from ten representative isolates. The genome-wide SNPs that were ascertained revealed the evolutionary history of CC121, indicating at least six major clades (A to F) within the clonal complex and dating its most recent common ancestor to the pre-antibiotic era. The toxin gene complement of CC121 isolates was correlated with their SNP-based phylogeny. Moreover, we found a highly significant association of clinical phenotypes with phylogenetic affiliations, which is unusual for *S. aureus*. All isolates evidently sampled from superficial infections (including staphylococcal scalded skin syndrome, bullous impetigo, exfoliative dermatitis, conjunctivitis) clustered in clade F, which included the European epidemic fusidic-acid resistant impetigo clone (EEFIC). In comparison, isolates from deep-seated infections (abscess, furuncle, pyomyositis, necrotizing pneumonia) were disseminated in several clades, but not in clade F. Our results demonstrate that phylogenetic lineages with distinct clinical properties exist within an *S. aureus* clonal complex, and that SNPs serve as powerful discriminatory markers, able to identify these lineages. All CC121 genomes harboured a 41-kilobase prophage that was dissimilar to *S. aureus* phages sequenced previously. Community-associated MRSA and MSSA from Cambodia were extremely closely related, suggesting this MRSA arose in the region.

## Introduction

*Staphylococcus aureus* is one of the most common human pathogens and causes a wide range of infections, from skin and soft tissue infections (SSTIs) to life threatening diseases such as pneumonia, endocarditis, bacteremia and toxic-shock syndrome [1], [2]. Among SSTI, two clinical entities predominate: (i) superficial infections that affect the upper skin layers exclusively (*stratum corneum* and *stratum granulosum*) and (ii) deep-seated infections associated with formation of abscesses such as furuncles, whitlows, and sweat-gland abscesses [3], [4]. Superficial infections are associated with formation of exfoliative toxins, most commonly exfoliative toxins A (ETA) and B (ETB) [5]–[9]. Exfoliative toxins are structurally related to a family of serine proteases and associated with the binding and cleavage of desmosomal cadherins, in particular desmoglein-1 (Dsg-1). The degradation of Dsg-1 provokes splitting of the granular cell layer in the epidermis, which causes blistering and exfoliation of the skin [5], [10]. The diseases associated with this mechansim are staphylococcal scalded skin syndrome (SSSS) and bullous impetigo. On the other hand, deep-seated infections are frequently caused by *S. aureus* strains that produce the Panton-Valentine leukocidin toxin (PVL) [4], [6], [11]. PVL is a bicomponent, pore-forming cytotoxin composed of two contiguous protein components (LukF-PV of 34 kDa and LukS-PV of 33 kDa). The destructive capability of PVL arises from the PVL-mediated lysis and apoptosis of polymorphonuclear leukocytes (PMNs or neutrophils) [12]. The two components LukF-PV and LukS-PV assemble into a pore-forming heptamer on the surface of PMNs, leading to PMN lysis and to the activation of several mechanisms (e.g. release of reactive oxygen species, inflammatory response) which contribute to tissue necrosis [13].

The association of the two different clinical manifestations of *S. aureus* infections with either exfoliative toxins ETA and ETB or the PVL toxin, respectively, is particularly well documented for *S. aureus* clonal complex CC121 [4], [14]–[16]. Complex CC121 is globally distributed, colonizing the anterior nares of asymptomatic subjects [14], [17]–[21]; however, it is also a common cause of both superficial skin infections [9], [14], [15] and deep-seated infections [4], [14]–[16]. Commonly, CC121 isolates are susceptible to methicillin. Recently, however, methicillin resistance had been found in CC121 among community-associated methicillin-resistant *S. aureus* (caMRSA) from Cambodia [19].

In the present study, we examined the population structure of CC121 on the basis of polymorphisms discovered in 115 genetic loci scattered around the staphylococcal genome. We reconstructed the evolutionary origin of caMRSA from PVL-producing methicillin-susceptible *S. aureus* in South-East Asia. We demonstrate that distinct phylogenetic lineages within CC121 are associated with specific courses of disease.

## Results and Discussion

### Global Population Structure

**Figure 1:**
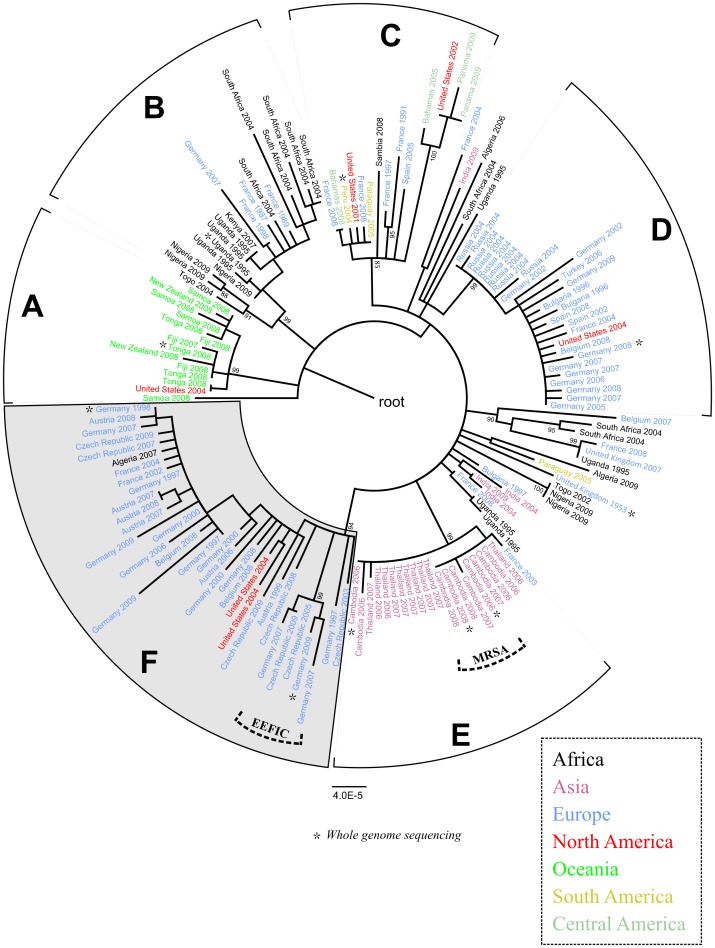
Phylogenetic relationships of 154 CC121 isolates. Maximum-likelihood phylogenetic tree based on 304 SNPs ascertained in 115 genetic loci (47 kb total) and annotated with the country of origin and year of isolation. The continent of origin is indicated by the color of the isolate's name: blue, Europe; orange, Asia; green, Oceania; red, North America; yellow, South America; black, Africa. Bootstrap values are shown where ≥85%. The scale bar represents substitutions per SNP site. Asterisks indicate isolates selected for whole-genome sequencing. The European epidemic fusidic acid-resistant impetigo clone (EEFIC) was identified on the basis of fusidic acid resistance, PCR detection of the *fusB* gene, *spa* typing (t171), and MLST (ST123).

We tested for sequence polymorphisms at 115 housekeeping loci of about 400 basepairs each (Table S1), in total covering about 1.7% (46,811 basepairs) of the genome from each of 154 *S. aureus* CC121 isolates. Among these isolates, 138 displayed sequence type ST121, the presumed ancestral type of clonal complex CC121 (Table S2). Eleven isolates displayed various single-locus variants of ST121, and five isolates displayed ST123, a double-locus variant (Table S2). Onehundred-fiftythree isolates had been sampled between 1987 and 2009 in 27 countries on five continents (Table S2). In addition, we included the phage-propagating strain PS71 from approximately 1953 [22]. We identified 304 single-nucleotide polymorphisms, 5 insertions and 7 deletions ranging in size from 1 to 12 basepairs (Table S3). SNPs included 93 synonymous base substitutions and 211 non-synonymous substitutions (Table S3). Based on these polymorphisms, our 154 isolates were assigned to 121 haplotypes (Figure 1). Ninety-two SNPs were parsimony-informative (i. e., the respective derived alleles were found in more than one haplotype) and the level of homoplasy was very low (homoplasy index, 0.03). The nucleotide diversity  =  was 0.00026±0.00001, which is nearly three times higher than the nucleotide diversity for *S. aureus* ST5 [23]. A SNP-based, maximum-likelihood phylogenetic analysis revealed at least six major clades, A to F, within *S. aureus* CC121 (Figure 1).

We applied a Bayesian coalescent method of phylogenetic inference implemented in the BEAST program [24] to estimate evolutionary rates and divergence times for CC121. Based on our set of DNA sequences from isolates that had been sampled between 1953 and 2009 (Table S2), we estimated the nucleotide substitution rate at 2×10^−6^ substitutions per nucleotide site per year (95% confidence intervals, 1.2×10^−6^ to 2.8×10^−6^). This short-term evolutionary rate is similar to rates previously reported for *S. aureus* [25], [26]. Based on this rate and the sequence variation observed in our dataset, the most recent common ancestor of CC121 was dated to 1884 (95% confidence interval, 1827 to 1925). This age estimate should be interpreted with caution because it is based on a relatively long extrapolation from available sequence data (note the large confidence interval). However, it is several fold longer than estimates available for MRSA [25]–[27], which might reflect the widespread endemic nature of *S. aureus* CC121 that precedes the antibiotic era.

The 154 isolates of our study displayed 38 different *spa* types, with six *spa* types covering 67% of all isolates (Figure S1). Some *spa* types were restricted to particular phylogenetic clades (t314 to clade B, t940 to clade C, t435 to clade D). However, the association of several other *spa* types (t284, t170, t272, t2155) with SNP-based phylogeny was imperfect, as each of them was found in several, unrelated haplotypes (Figure S2). The maximum monophyletic clade (MC) statistics calculated for these *spa* types by using BaTS software [28] confirmed the absence of significant associations with phylogeny, because they were not significantly different from MC values for a randomized, null distribution (Table S4). These results suggested *spa* homoplasies, similar to previous reports on other *S. aureus* sequence types [23], [25], [29].

### Phylogeography

The isolates' phylogenetic relationships reflect their geographic origin to a considerable extent, suggesting that specific subclones of CC121 are endemic in the regions sampled (Figure 1). Accordingly, for several geographic regions, Bayesian analysis with BaTS software indicated associations of geographic origins with phylogeny (Table S4). For example, clade A encompasses all 12 isolates from Oceania, including strains from New Zealand, Samoa, Tonga and Fiji. Similarly, clade C encompasses six out of seven isolates from South America, including Peru, Panama, Paraguay and the Bahamas. Further, all 20 isolates in clade E are from Southeast-Asia, including Thailand and Cambodia, clade B contains most of the isolates from Africa (Nigeria, Uganda, Togo, South Africa and Kenya), and clades D and F contain isolates mostly from Europe. Isolates from Europe are comparatively more diverse (Figure 1), possibly due to more extensive sampling coverage from this region, as 45% of the isolates originate from Europe.

The epidemic European fusidic acid-resistant impetigo clone (EEFIC), which is characterized by exfoliative toxin production, the *fusB* gene and, most commonly, *spa* type t171 [15], is a subclone of clade F (Figure 1). Evidently, EEFIC has spread clonally throughout several European countries [30], [31], and here we report that EEFIC has been present also in Germany and the Czech Republic since at least 2007 and 2005, respectively (Table S2).

Our analysis included five isolates of ST121 community-associated MRSA from Cambodia, which had been reported only recently [19], [32]. These MRSA isolates are very closely related to each other and to MSSA from the same area, displaying a maximum distance of only one point mutation in the 47 kb of DNA analysed (Figure 1). This result suggests that ST121 MRSA arose locally in Cambodia (and only once), which had been assumed previously based on the high prevalence of methicillin-susceptible *S. aureus* (MSSA) ST121 in the country and similar antibiotic resistance patterns among MRSA ST121 and MSSA ST121 from the same geographic region [19].

### Comparative Genomics

**Figure 2:**
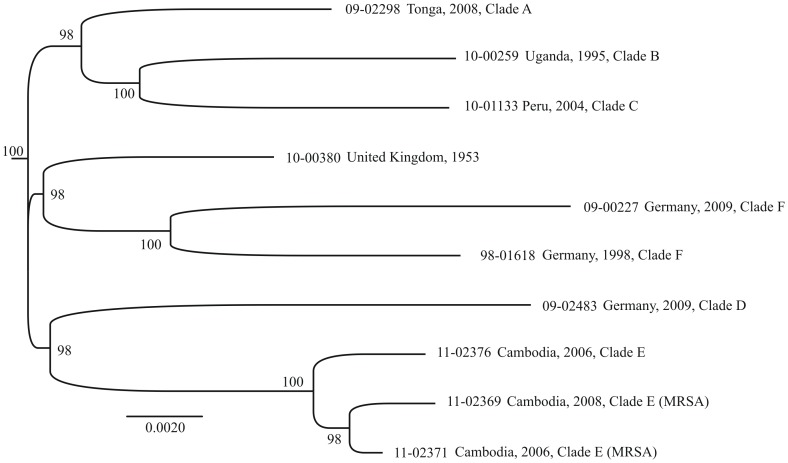
Maximum-likelihood phylogenetic tree based on an alignment of 1,828 SNPs ascertained in 2,209 kb from pyro-sequenced genomes. Bootstrap values >90% are indicated.

We pyro-sequenced the genomes from ten CC121 isolates, including the oldest isolate in the collection, which had been collected in 1953, and one to three isolates from each of the phylogenetic clades A to F (Figure 1). Genome-wide SNPs from these selected isolates were ascertained by mapping sequencing reads onto an ST5 reference sequence by using SSAHA2 software. These SNPs largely confirmed the phylogenetic relationships that had been revealed from 47-kb sequences from the larger set of isolates, including the relatedness of MRSA and MSSA from Cambodia (Figure 1, Figure 2). However, genome sequences provided more precise estimates of internodal distances and indicated that clades A, B, and C are related and clades D and E are related (Figure 2), which had not been resolved on the basis of more limited data (Figure 1). Genome sequences provided no evidence of recombination (homoplasy index, 0.05; no recombination detected with Recombination Detection Program RDP 3.44). In addition, sequencing reads were assembled *de-novo* by applying Newbler software, revealing the gene content for each of the sequenced genomes (Table S6). All CC121 isolates harboured a 41-kb prophage that was dissimilar from *S. aureus* phages sequenced previously with respect to DNA sequence, gene content, integrase group, and capsid-gene based phage type (Table S5). The prophage encoded 55 open-reading frames, most of which could not be assigned any specific functions based on sequence similarity (Figure S3). CC121 genomes contained one to three additional prophages, including an integrase group *Sa1* phage encoding the *eta* gene or a group *Sa2* phage encoding the PVL gene (Table S6). Each genome had three pathogenicity islands (SaPI), with strain-specific variation (Table S6). MRSA carried a methicillin-resistance island that shared >99% sequence similarity with previously reported SCC*mecV* (sequence accession number GQ902038 [33]).

Because methicillin-resistant CC121 had not been found until recently, we investigated whether CC121 genomes may lack the DNA sequences necessary for integration of the SCC*mec* element (the genetic determinant of methicillin resistance in staphylococci). SCC*mec* integrates into a specific attachment site (*attB*) at the 3'-end of a gene named *orfX* in the staphylococcal chromosome, and it has been proposed that the core of the attachment site itself and the 100 basepairs left from it need to be highly conserved to enable efficient insertion or excision of the SCC*mec* element [34]. In the ten genomes we have sequenced, this chromosomal region was identical to that from another ST121 isolate [34], except for a single point mutation in two ST121-MRSA and their close, methicillin-susceptible relative from Cambodia, indicating that the *attB* sequence in CC121 does not prevent SCC*mec* integration. It has also been proposed that a presumptive mobile genetic element encoding an enterotoxin gene (*seh*) interferes with SCC*mec* motility, when inserted downstream of the SCC*mec* attachment site [35]. All of our CC121 genomes had a similar element at this position, encoding a different enterotoxin gene. However, this element was also present in ST121-MRSA, so it does not appear to hinder integration of SCC*mec* in CC121.

### Correlation of Clinical Phenotype and Toxin Gene Complement with Phylogeny

**Figure 3:**
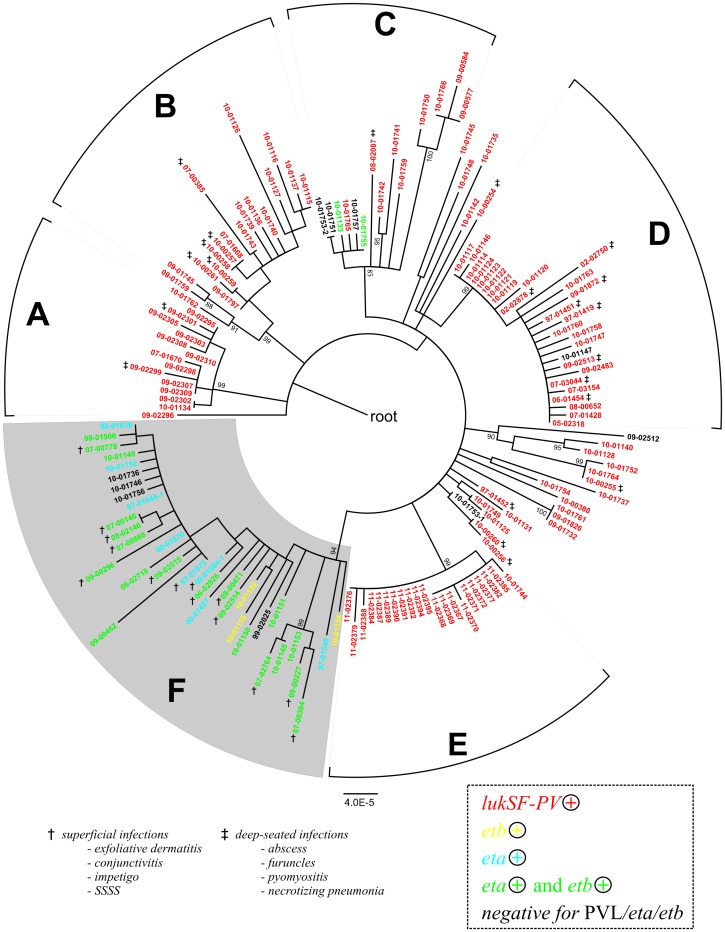
Toxin gene complement and clinical phenotype. Maximum likelihood phylogenetic tree as in Figure 1, indicating the presence of toxin genes *lukSF-PV*, *eta*, and *etb* (colours) and clinical phenotype where this was reported (symbols: **†**, superficial infection, i. e., impetigo, staphylococcal scalded skin syndrome, conjunctivitis, or exfoliative dermatitis); ‡, deep-seated infection (abscess, furuncle, pyomyositis, necrotizing pneumonia).

Bayesian analysis by using BaTS software indicated that the isolate toxin gene complement was strongly associated with their SNP-based phylogeny, because the MC values calculated for the presence of the toxin genes, the association index (AI) and the parsimony score (PS) all were significantly different from statistics estimated for a null distribution; p≤0.01; Table S4). Clade F encompassed 35 isolates, 21 of which carried the exfoliative toxin genes *eta* and *etb*, ten carried either one of the two, and four tested negative for both, *eta* and *etb* (Figure 3). We noted that three clade F isolates (97-01548, 97-01848-1, 00-01004-1) were *etb*-negative now, even though they had been tested *etb*-positive several years ago according to records in our strain collection database, presumably reflecting the instability of the *etb* carrying plasmid during long-term storage or sub-cultivation. None of the isolates in clade F tested positive for the *lukS/F* gene encoding the PVL toxin. In contrast, isolates outside of clade F were preferentially linked with the presence of the *lukS/F* genes (Figure 3). Upon sequencing the *lukS/F* genes, we identified sequence variants R, H1, H2, and H3, all of which had been reported previously to be present in multiple other clonal complexes of *S. aureus* (24), plus six novel sequence variants (Table S7), indicating that CC121 has acquired the *lukS/F*-carrying phage multiple times.

It is not clear why *lukS/F* and *eta/etb* genes are mutually exclusive in CC121. *LukS/F* and *eta* reside on unrelated prophages that integrate at different positions into the genome, and *etb* is commonly encoded by a plasmid, whose mode of interaction with prophages is not known. We noted that one of the genomes from clade F (isolate 09-00227 with *eta*) lacked the attachment site for the *lukS/F*-carrying phage [36] due to a deletion event. However, by using a dedicated PCR (Table S8), we found that this deletion was not a common feature of clade F isolates. Rather, the attachment site was present in all clade F isolates except for those affiliated to the EEFIC clade (Figure 1) and one other isolate. It is possible that the observed association between gene complement and phylogeny is driven simply by the extensive international spread of clonal strains that carry one or other toxin genes, rather than any mechanistic hindrances to gene acquisition (Figure 1, Figure 3). For example, antibiotic usage promotes the spread of resistant *S. aureus* strains, but in our sample of CC121 no specific resistance traits were associated with clade F (Table S2). Reportedly, *Staphylococcus aureus* strains with exfoliative toxins frequently also produce EDIN exotoxins [37]. Because the exact role of EDIN in staphylococcal infections has not yet been explored, this issue was not followed here.

We were able to ascertain the type of disease associated with 46 of the isolates in our international CC121 collection. Of these, 35 infections could be unequivocally classified as either superficial or deep-seated, whereas 11 were wound infections or unspecified 'skin and soft tissue infections' (Table S2). Strikingly, all isolates associated with superficial infections (n = 14) (including SSSS, bullous impetigo, exfoliative dermatitis, and one case of conjunctivitis) clustered in clade F (Figure 3). In contrast, isolates from deep-seated infections (n = 21) (abscesses, furuncles, pyomyositis, necrotizing pneumonia) were disseminated in several clades, but not in clade F (Figure 3). Accordingly, the association of the clinical phenotype with the phylogenetic affiliation of the respective isolates was highly significant (i. e., MC, AI, and PS statistics were significantly different from those for a null distribution; p≤0.01; Table S4).

Links between specific types of disease and phylogenetic groupings within the species *S. aureus* have been highly unusual findings in the past. For example, MLST-defined clonal lineages of *S. aureus* generally do not correlate with virulence properties or with any other phenotypic traits that may be of interest to clinicians [38]. Our results demonstrate that *S. aureus* strains with specific clinical properties exist within clonal complexes, and that SNPs may be powerful discriminatory markers to identify them (Table S9). In the light of accelerated and abundant pathogen genome sequencing [25], we anticipate that additional *S. aureus* strains with association to specific clinical symptoms may be recognized in the future, and that (SNP-based) typing could eventually be used to predict the pathogenic potential of a given isolate. In many cases, however, the propensity to cause a specific clinical picture will depend on interactions between multiple proteins rather than a single toxin.

## Methods

### Bacterial Isolates

The sources and characteristics of *S. aureus* isolates are provided in Table S2. Some of these isolates had been included in previous studies as indicated in Table S2 [16], [19], [22], [32], [39]. To represent global diversity, we included 154 isolates from 27 countries on five continents. Antimicrobial susceptibility was tested by using the microbroth dilution method according to DIN58940 and applying the EUCAST breakpoints. *Spa*-typing was performed according to the Ridom StaphType standard protocol (www.ridom.org) and *spa*-types were assigned using the respective StaphType software (Ridom GmbH, Würzburg, Germany). MLST was performed as described previously [40]. Primers for the detection of *eta*-, *etb*- and *lukSF-PV*-genes are summarized in Table S8. The different *lukSF-PV* sequence variants were determined as specified by ÓHara *et al.* [41].

### Mutation Discovery by dHPLC

Mutation discovery was performed as described previously [23], [42]. Briefly, PCR-amplified gene fragments were analyzed by dHPLC (WaveR Nucleic Acid Fragment Analysis System, Transgenomic). The 115 genetic loci investigated, PCR primers, and dHPLC conditions are listed in Table S1. Subsequently, mutations were verified in all affected isolates by capillary sequencing. Discovered polymorphisms and their properties are summarized in Table S3.

### Data Analysis

Sequences from 115 loci were concatenated for each of 154 isolates, resulting in a 46,811-bp sequence alignment. A maximum likelihood tree based on this alignment was constructed with PhyML 3.0.1. The ancestral node was determined by including the genome sequence from distantly related *S. aureus* N315 (ST5; GenBank accession number BA000018). DnaSP was used to calculate the mean pair-wise distance between alleles at synonymous (Ks) and non-synonymous (Ka) sites and to estimate the nucleotide diversity (π) and nucleotide variation (θw) [43]. The homoplasy index was estimated with Paup 4.0. Short-term evolutionary rates and divergence dates were estimated by applying the BEAST software, version 1.6.2 (http://beast.bio.ed.ac.uk/) [24], dating sequences with the year of isolate sampling, and running 10^8^ iterations after a burn-in phase of 10^6^ iterations. BEAST results were virtually independent from applied clock models (strict, relaxed) and tree priors (constant population size, Bayesian skyline).

We applied Bayesian tip-association significance testing (BaTS, version 1.0; [28]) to assess phylogeny-trait associations for clinical phenotypes, toxin gene complement, *spa* types, geographic origin, and antimicrobial resistance, each based on sets of 100 trees sampled from BEAST results. In each case, isolates for which the respective trait information was not available had been excluded from the initial sequence alignments. The association index (AI), the parsimony score (PS), and the maximum monophyletic clade (MC) statistics were computed [28]. To avoid any inflation of the statistical significance of associations due to multiple, indistinguishable isolates in the dataset, calculations were repeated after excluding redundant isolates, and both sets of statistics are reported (Table S4). The null hypothesis of no association between phylogeny and traits was estimated on the basis of 100 randomizations, and the null hypothesis was rejected if p<0.05 (Table S4).

### Genome Sequencing

Staphylococcal genome sequences were generated commercially (GATC, Konstanz, Germany) on a 454 FLX machine, applying FLX+ chemistry and resulting in 12- to 21-fold average coverage. De-novo assemblies with 454 Newbler software resulted in 68 to 156 contigs (>500 bp) per genome. Sequence annotation was performed by comparisons to previously published *S. aureus* genomes, applying the annotation tool implemented in Kodon software (Applied Maths), and by BLAST searches in the GenBank database (http://blast.ncbi.nlm.nih.gov/Blast.cgi). In addition, prophage sequences were identified with PHAST (phage search tool, available at http://phast.wishartlab.com/ [44]). Genome-wide SNPs were ascertained by mapping the sequencing reads onto the N315 genome sequence (GenBank accession number BA000018), applying SSAHA2 software (available at http://www.sanger.ac.uk/resources/software/ssaha2/) and filtering the resulting output for SNPs with a minimum consensus quality of 30, a minimum mapping quality of 30, and a minimum coverage of 5. SNPs in mobile genetic elements (SaPIs, prophages, transposons, IS elements), repetitive regions, homopolymeric regions, and within 100 bp from contig ends were removed manually, resulting in a dataset of 1,828 SNPs ascertained in the core genome of 2,208,736 basepairs. An alignment of core genome sequences from ten CC121 isolates and from the reference N315 was used to reconstruct a maximum likelihood phylogenetic tree with PhyML3.01. The Recombination Detection Program (RDP 3.44, [45]) was applied to screen the alignment for evidence of recombination based on the RDP, Geneconv, Chimaera, MaxChi, Bootscan, and SiScan methods (see RDP manual), applying default parameters and p<0.05.

Sequence data has been deposited at the NCBI Sequence Read Archive (http://www.ncbi.nlm.nih.gov/sra) under accession numbers SRX209921 to SRX209927, SRX208966, and SRX209760.

## Supporting Information

Figure S1 **Distribution of the six major** ***spa*** **-types.** Maximum likelihood phylogenetic tree based on 304 SNPs from a selection of housekeeping genes annotated with the respective *spa*-types, indicated by the following colors: red, t159; light blue, t284; green, t314; yellow, t435; blue, t645; magenta, t940.
(PDF)

Figure S2 **Distribution of homoplasious** ***spa*** **-types.** Maximum likelihood phylogenetic tree based on 304 SNPs from a selection of housekeeping genes annotated with the respective *spa*-types.
(PDF)

Figure S3 **Prophage ΦSaCC121.** Prophage modules are color coded: lysogeny, red; DNA replication, orange; transcriptional regulation, yellow; DNA packaging and head, green; tail, blue; lysis, magenta; hypothetical proteins, black. Selected genes are indicated: int, integrase; rep, repressor; p.rep*, putative repressor HTH protein; ant, antirepressor; hel, helicase; pol, polymerase; pri, primase; terS/L, small and large subunit terminase; pro, portal; mhp, major head protein; tape, tape measure protein (tmp); hol, holin; ami, amidase.
(PNG)

Table S1 **Genetic loci and PCR primers used for WAVE analysis.**
(XLS)

Table S2 **Bacterial isolates.**
(XLS)

Table S3 **Polymorphisms discovered in the genome fragments from 154 isolates.**
(XLS)

Table S4 **Results of Bayesian tip-association significance testing.**
(XLSX)

Table S5 **Predicted ORFs in ST121 Phage.**
(XLSX)

Table S6 **Genome content.**
(XLSX)

Table S7 **PVL nucleotide variation.**
(XLS)

Table S8 **PCR primers.**
(XLS)

Table S9 **Mutations defining clades within CC121.**
(XLSX)
