# Supplementary figures and images for: Subpopulations of Staphylococcus aureus Clonal Complex 121 Are Associated with Distinct Clinical Entities
Source: PLoS One. 2013 Mar 7;8(3):e58155. doi: 10.1371/journal.pone.0058155 (PMC3591430; doi:10.1371/journal.pone.0058155)

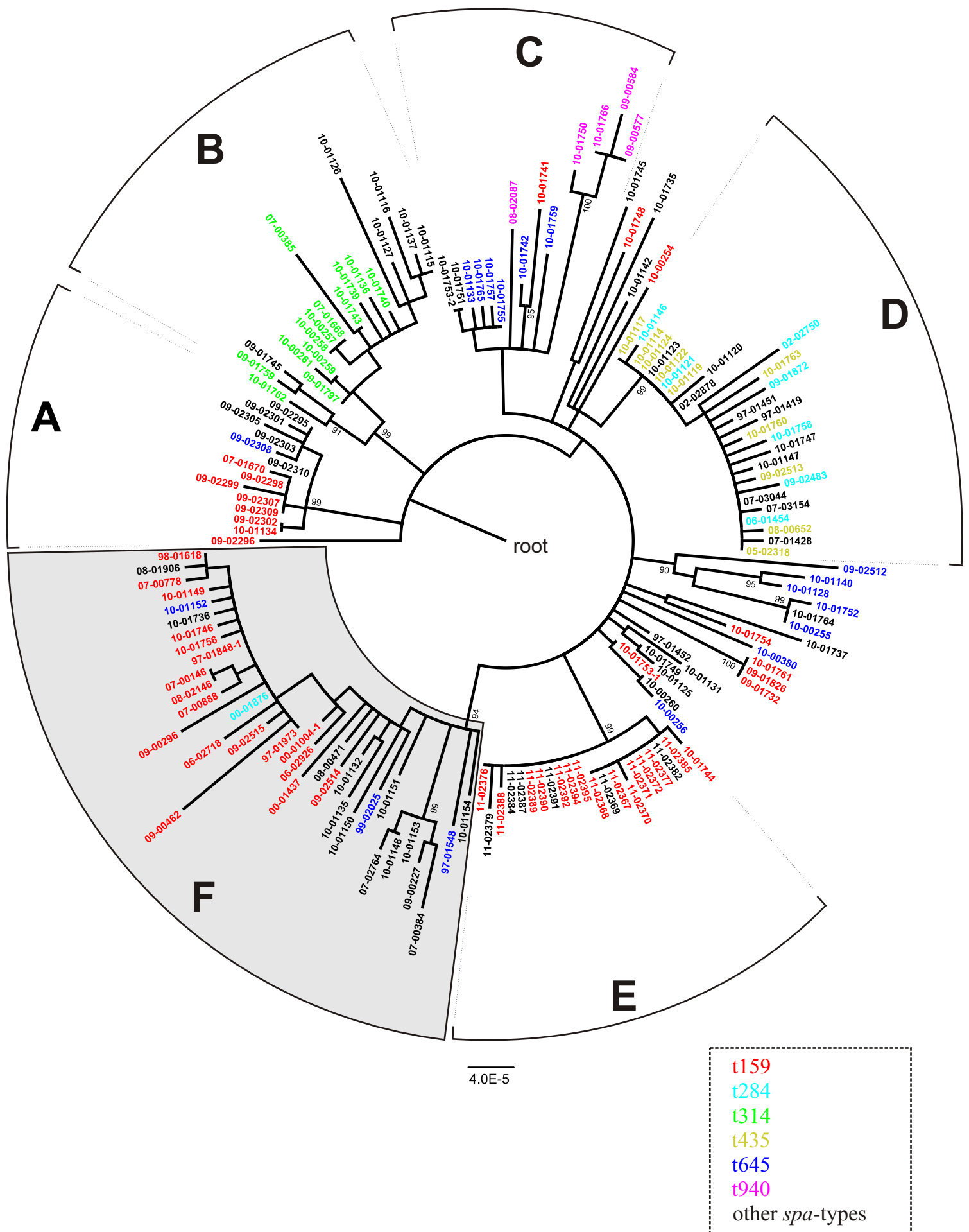

**Figure S1**

Supplement: Figure S1 — Distribution of the six major spa -types. Maximum likelihood phylogenetic tree based on 304 SNPs from a selection of housekeeping genes annotated with the respective spa-types, indicated by the following colors: red, t159; light blue, t284; green, t314; yellow, t435; blue, t645; magenta, t940. (PDF) [file pone.0058155.s001.pdf]

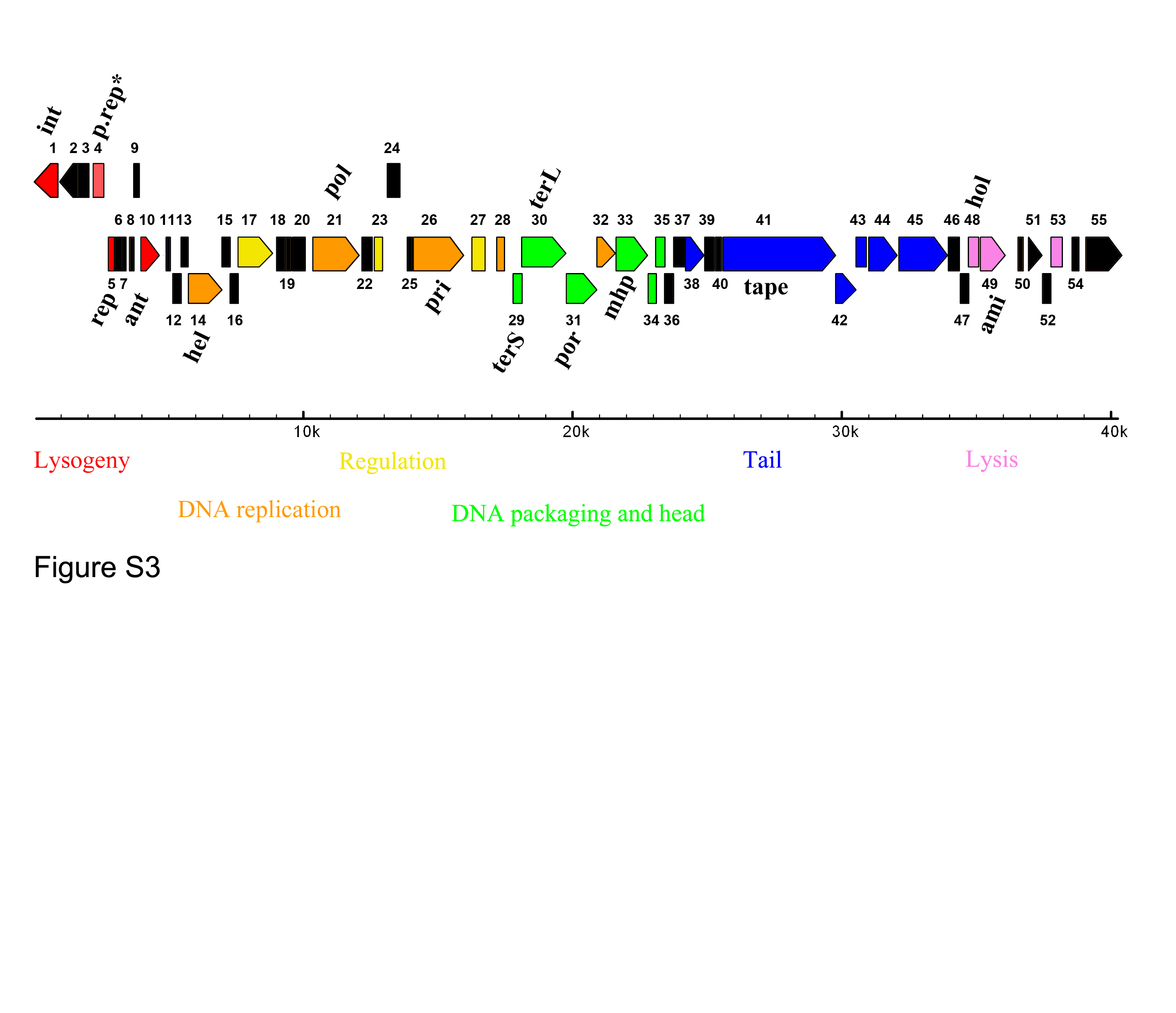

Supplement: Figure S3 — Prophage ΦSaCC121. Prophage modules are color coded: lysogeny, red; DNA replication, orange; transcriptional regulation, yellow; DNA packaging and head, green; tail, blue; lysis, magenta; hypothetical proteins, black. Selected genes are indicated: int, integrase; rep, repressor; p.rep*, putative repressor HTH protein; ant, antirepressor; hel, helicase; pol, polymerase; pri, primase; terS/L, small and large subunit terminase; pro, portal; mhp, major head protein; tape, tape measure protein (tmp); hol, holin; ami, amidase. (PNG) [file pone.0058155.s003.png]
