# Supplementary material for: Subpopulations of Staphylococcus aureus Clonal Complex 121 Are Associated with Distinct Clinical Entities
Source: PLoS One. 2013 Mar 7;8(3):e58155. doi: 10.1371/journal.pone.0058155 (PMC3591430; doi:10.1371/journal.pone.0058155)
Supplement: Figure S2 — Distribution of homoplasious spa -types. Maximum likelihood phylogenetic tree based on 304 SNPs from a selection of housekeeping genes annotated with the respective spa-types. (PDF) [file pone.0058155.s002.pdf]

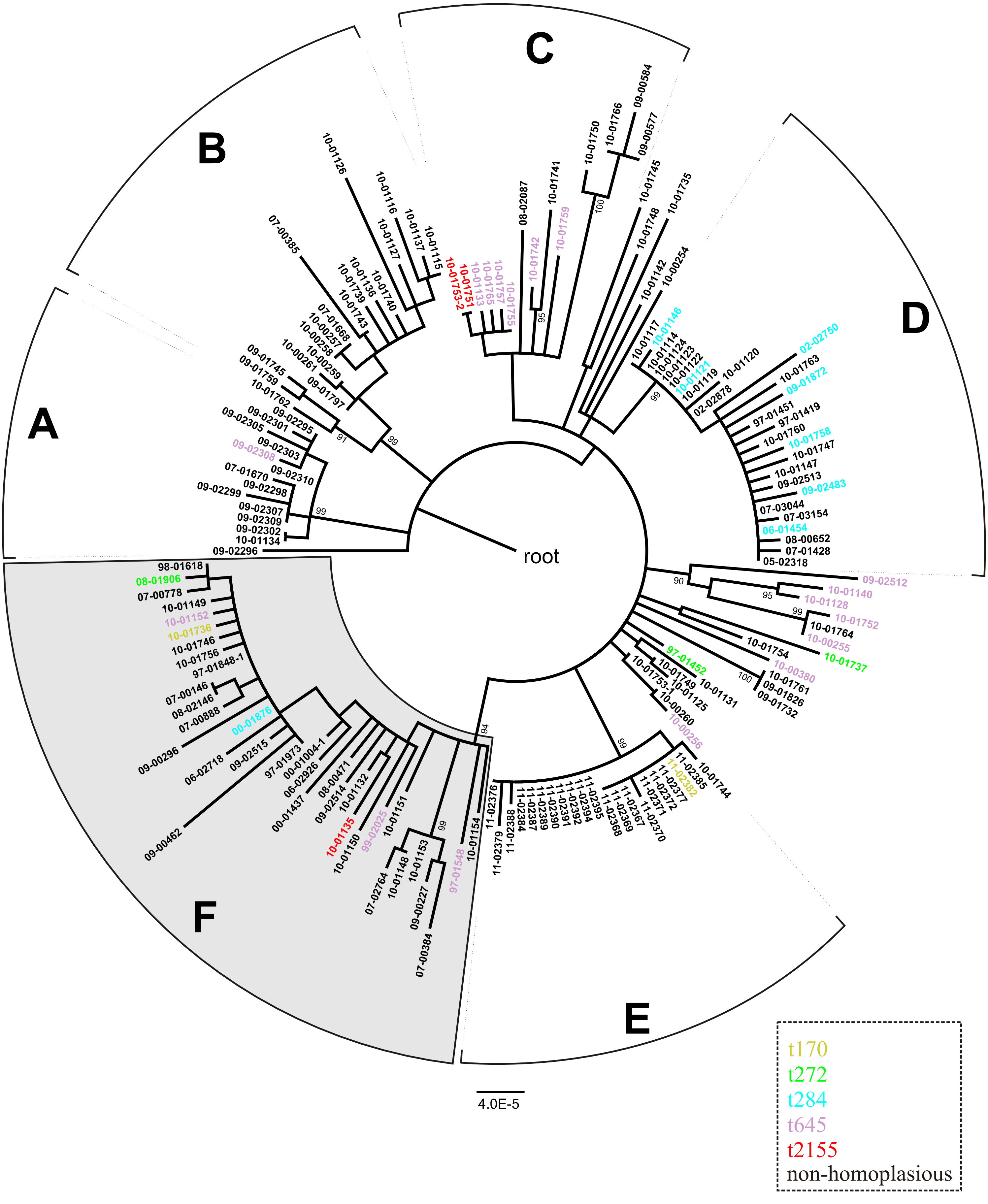
A
B
C
D
E
F
root
09-01745
09-01759
10-01762
09-02301
09-02305
09-02295
09-02308
09-02303
07-01670
09-02310
09-02299
09-02298
09-02307
09-02309
09-02302
10-01134
09-02296
10-01126
10-01116
10-01127
10-01137
10-01115
07-00385
10-01136
10-01739
10-01740
10-01743
07-01668
10-00257
10-00258
10-00261
10-00259
09-01797
10-01753-2
10-01751
10-01133
10-01765
10-01757
10-01755
10-01742
10-01759
08-02087
10-01741
10-01750
10-01766
09-00584
09-00577
10-01745
10-01735
10-01748
10-01142
10-00254
10-01117
10-01146
10-01114
10-01124
10-01123
10-01122
10-01121
10-01119
02-02878
10-01120
02-02750
10-01763
09-01872
97-01451
97-01419
10-01760
10-01758
10-01747
10-01147
09-02513
09-02483
07-03044
07-03154
06-01454
08-00652
07-01428
05-02318
09-02512
10-01140
10-01128
10-01752
10-01764
10-00255
10-01754
10-01737
10-00380
10-01761
09-01826
09-01732
97-01452
10-01749
10-01131
10-01753-1
10-01125
10-00260
10-00256
10-01744
11-02385
11-02382
11-02377
11-02372
11-02371
11-02370
11-02367
11-02369
11-02368
11-02395
11-02394
11-02392
11-02391
11-02390
11-02389
11-02387
11-02384
11-02388
11-02379
11-02376
10-01154
98-01618
08-01906
07-00778
10-01149
10-01152
10-01736
10-01746
10-01756
97-01848-1
07-00146
08-02146
07-00888
00-01876
09-00296
06-02718
09-02515
97-01973
00-01004-1
06-02926
00-01437
08-00471
09-02514
10-01132
10-01135
10-01150
99-02025
10-01151
07-02764
10-01148
10-01153
09-00227
07-00384
97-01548
09-00462
99
91
99
95
100
99
90
95
99
100
99
94
99
4.0E-5
t170
t272
t284
t645
t2155
non-homoplasious

Figure S2
